# Early Retinal Microvascular Alterations in Prediabetes and Diabetes Without Clinically Detectable Retinopathy

**DOI:** 10.5152/eurasianjmed.2026.261459

**Published:** 2026-08-28

**Authors:** Onur Furundaoturan, Cumalı Değirmenci

**Affiliations:** 1 Department of Ophthalmology, andırma On Yedi Eylül University, Balıkesir, Türkiye; 2 Department of Ophthalmology, Ege University, İzmir, Türkiye

Dear Editor,

We read with interest the article by Utlu et al^1^ evaluating foveal avascular zone (FAZ) characteristics and macular vessel density in prediabetic patients using optical coherence tomography angiography (OCTA). The study addresses an increasingly relevant question and adds to the growing literature on early retinal microvascular alterations in metabolic disease. We would like to contribute a comparative perspective drawn from our recent work in diabetic patients without retinopathy^2^ and to raise several methodological considerations.

Utlu et al^1^ reported reduced Superficial Capillary Plexus (SCP) and deep capillary plexus (DCP) perfusion density in prediabetic eyes, reaching significance in the temporal quadrant of the SCP and the inferior and temporal quadrants of the DCP, together with a non-significant trend toward FAZ enlargement. In our cohort of 95 eyes of patients with type 2 diabetes without retinopathy and 90 control eyes, vascular density was significantly lower in both plexuses across all measured regions, and the FAZ area was significantly larger in patients than in controls (0.285 ± 0.147 mm^2^ vs. 0.259 ± 0.105 mm^2^, *P* = .029), with shape irregularities present in 37% versus 10% of eyes (*P* < .001).^2^ Hemoglobin A1c (HbA1c) correlated positively with FAZ area (*r* = 0.26, *P* = .012), whereas Utlu et al^1^ reported a strong negative correlation between HbA1c and DCP vascular density. The fact that a different parameter tracked glycemic status in each study underlines how device, segmentation, and cohort characteristics shape OCTA results.

These differences should be interpreted in light of the methodological distinctions. The studies differ in sample size (60 vs. 185 eyes), OCTA platform (Nidek vs. Optovue), scan protocol, and patient selection, all of which can affect quantitative OCTA metrics and contribute to the heterogeneity reported in this field,^3^ thereby limiting direct numerical comparison. Moreover, early retinal microvascular change is unlikely to reflect glycemic status alone. Diabetes duration, hypertension, dyslipidemia, body mass index, smoking, and renal function may all influence OCTA parameters; these variables were only partly controlled and differed between the cohorts (e.g., mean age was approximately 41 years in the prediabetic group versus 54 years in our diabetic group), and they should be regarded as potential confounders.

Taken together, the 2 studies are consistent with the hypothesis that retinal microvascular impairment may begin in the prediabetic stage and become more pronounced in established diabetes before clinically detectable retinopathy. However, because the cohorts were studied separately, with different devices and protocols, this progression cannot be confirmed by cross-study comparison and should be tested in prospective studies that evaluate prediabetic and diabetic groups concurrently using a standardized protocol.

In conclusion, OCTA is a promising non-invasive tool for detecting subclinical retinal microvascular changes in the prediabetic and pre-retinopathic stages. Before it can be adopted as a clinical screening tool, however, prospective and standardized studies are required to establish reproducible thresholds and to account for the metabolic and systemic factors that influence the retinal microvasculature.

## Data Availability

The data that support the findings of this study are available on request from the corresponding author.
